# Health technology in primary health care: a path to promote health care delivery

**DOI:** 10.26633/RPSP.2019.48

**Published:** 2019-05-24

**Authors:** Bryan Valcarcel

**Affiliations:** 1 Escuela de Medicina Humana Escuela de Medicina Humana Universidad Científica del Sur Lima Perú Escuela de Medicina Humana, Universidad Científica del Sur Lima, Perú

Dear Editor:

In recent years, Peru has had pivotal health achievements. For example, for the population overall, coverage with either private or public health insurance increased from 37% to 76% over a 13-year period (1). In addition, for the period of 2016–2017, the mortality rate for children under 5 years old and for neonates was, respectively, 18 and 10 deaths per 1 000 newborns, both of which were below the corresponding Sustainable Development Goals thresholds, of 25 and 12 deaths per 1 000 newborns (2). Nevertheless, a recent report pointed out that chronic noncommunicable diseases (NCDs) are the leading cause of mortality in approximately 50% of the population (1). Peru’s demographic and epidemiological transition has led to improvements in some health care indicators. However, the shift toward higher levels of NCDs in the population poses new public health challenges for health care practitioners, policymakers, and the country overall, for which new solutions are needed.

One strategy to promote and deliver health care is the implementation and use of health technology, which the World Health Organization (WHO) defines as the use of systematic and organized knowledge in the form of devices, medicines, vaccines, procedures, and systems. The WHO and the United Nations Children’s Fund cohosted the Global Conference on Primary Health Care in Kazakhstan on 25 and 26 October 2018. At that meeting, the participants endorsed the Declaration of Astana, which called for promoting and recommitting to the delivery of high-quality primary care around the world. For that to happen, the Declaration stated, it is essential to implement technology to build sustainable primary health care and achieve universal health coverage (3).

As part of that effort to promote primary health care, using mobile health (mHealth) technology, such as smartphones, could help reduce NCD morbidity and mortality in the population. In Peru, some community public health experiences with this new technology have produced promising results. For example, Diez-Canseco et al. (4) developed and conducted the Allillanchu Project, with the aim of using mHealth technology to screen for mental disorders in a low-income community. The project involved three types of public health workers (nurses, nurse assistants, and midwives) in five different health facilities. During their routine work activities, these workers screened 733 patients over a nine-week period. Of those 733, 159 (21.7%) were found positive in that assessment, and 127 (17.3%) attended a follow-up interview. These 127 patients received positive messages and reminders to seek specialized care, via text messages sent to their cell phones. Of these 127, 92 of them (72.4%) reported seeing a specialist.

In another investigation of new technology, Rubinstein et al. (5) used mobile phones to promote healthy lifestyle practices in low-resource urban settings in Peru and two other countries of Latin America. In their randomized controlled trial, those researchers assigned 637 patients with prehypertension to receive either conventional care (only receiving a leaflet about healthy lifestyles) or personalized monthly motivational cell phone calls and weekly text messages. In contrast to the control group, the patients in the mHealth intervention group reduced their body weight and increased their consumption of fruits and vegetables.

Health technology is a still-evolving field in Peru. These two examples show how mHealth can provide new approaches for diagnosing and reducing morbidity from NCDs. In order to fully benefit from mHealth, Peru needs a collective effort that involves broad-based support from governmental sectors and private organizations, as well as incorporates community feedback. I am positive that health technology will reform health delivery in Peru and will be a driving force to make the health care sector more sustainable.

## Disclaimer.

The author holds sole responsibility for the views expressed in the manuscript, which may not necessarily reflect the opinion or policy of the *RPSP/PAJPH* and/or PAHO.

**Bryan Valcarcel**

Escuela de Medicina Humana, Universidad Científica del Sur

Lima, Perú

bryan.valcarcel@gmail.com
